# Discovery and Functional Annotation of SIX6 Variants in Primary Open-Angle Glaucoma

**DOI:** 10.1371/journal.pgen.1004372

**Published:** 2014-05-29

**Authors:** Megan Ulmer Carnes, Yangfan P. Liu, R. Rand Allingham, Benjamin T. Whigham, Shane Havens, Melanie E. Garrett, Chunyan Qiao, NEIGHBORHOOD Consortium Investigators, Nicholas Katsanis, Janey L. Wiggs, Louis R. Pasquale, Allison Ashley-Koch, Edwin C. Oh, Michael A. Hauser

**Affiliations:** 1 The Center for Human Genetics, Duke University, Durham, North Carolina, United States of America; 2 The Center for Human Disease Modeling, Duke University Medical Center, Durham, North Carolina, United States of America; 3 Department of Ophthalmology, Duke University Medical Center, Durham, North Carolina, United States of America; 4 Department of Ophthalmology, Harvard Medical School, Boston, Massachusetts, United States of America; 5 Beijing Tongren Hospital, Beijing Tongren Eye Center, Beijing Ophthalmology & Visual Sciences Key Laboratory, Capital Medical University, Beijing, China; 6 Channing Division of Network Medicine, Brigham and Women's Hospital, Boston, Massachusetts, United States of America; 7 Department of Medicine, Duke University Medical Center, Durham, North Carolina, United States of America; 8 Department of Neurology, Duke University Medical Center, Durham, North Carolina, United States of America; Georgia Institute of Technology, United States of America

## Abstract

Glaucoma is a leading cause of blindness worldwide. Primary open-angle glaucoma (POAG) is the most common subtype and is a complex trait with multigenic inheritance. Genome-wide association studies have previously identified a significant association between POAG and the *SIX6* locus (rs10483727, odds ratio (OR) = 1.32, p = 3.87×10^−11^). *SIX6* plays a role in ocular development and has been associated with the morphology of the optic nerve. We sequenced the *SIX6* coding and regulatory regions in 262 POAG cases and 256 controls and identified six nonsynonymous coding variants, including five rare and one common variant, Asn141His (rs33912345), which was associated significantly with POAG (OR = 1.27, p = 4.2×10^−10^) in the NEIGHBOR/GLAUGEN datasets. These variants were tested in an *in vivo Danio rerio* (zebrafish) complementation assay to evaluate ocular metrics such as eye size and optic nerve structure. Five variants, found primarily in POAG cases, were hypomorphic or null, while the sixth variant, found only in controls, was benign. One variant in the *SIX6* enhancer increased expression of *SIX6* and disrupted its regulation. Finally, to our knowledge for the first time, we have identified a clinical feature in POAG patients that appears to be dependent upon *SIX6* genotype: patients who are homozygous for the *SIX6* risk allele (His141) have a statistically thinner retinal nerve fiber layer than patients homozygous for the *SIX6* non-risk allele (Asn141). Our results, in combination with previous SIX6 work, lead us to hypothesize that *SIX6* risk variants disrupt the development of the neural retina, leading to a reduced number of retinal ganglion cells, thereby increasing the risk of glaucoma-associated vision loss.

## Author Summary

Primary open angle glaucoma is a blinding disease for which there is currently no cure, only treatments that may slow its progress. To help understand the mechanisms of this disease and to design more effective treatments, we identified previously a locus, *SIX6*, that increases the risk of glaucoma. This gene is involved in early eye development and helps to form the retina. In this paper, we test specific sequence variants in *SIX6* that are found in glaucoma patients. We show that these variants have a reduced function that interferes with their ability to direct proper formation of the retina. One variant in particular is common, and may be the main reason that this gene is important in the glaucoma disease process. Patients who have two copies of this sequence variant show a change in the structure of their eye consistent with fewer neurons that carry the visual signal to the brain. These neurons typically die as people age, and people who begin life with fewer visual neurons may have an increased risk of glaucoma. Additional research in this topic may lead to new treatments that preserve sight.

## Introduction

Primary open-angle glaucoma (POAG) is the most common type of glaucoma, a group of diseases that affect approximately 60 million people worldwide and is a leading cause of blindness [1]. Glaucoma is characterized by the progressive death of retinal ganglion cells, leading to optic nerve atrophy and loss of vision. POAG is a complex inherited disorder for which an increasing number of genetic associations have been described, each contributing modestly to disease burden [2], [3].

A recent POAG genome-wide association study found a significant genetic association (rs10483727, odds ratio (OR) = 1.32, p = 3.87×10^−11^) at the *SIX1/SIX6* locus [4]. Variants in the *SIX1/SIX6* locus were first associated with quantitative optic nerve parameters in controls, including vertical cup-disc ratio (VCDR), which is used clinically to diagnose and monitor POAG progression [5], [6]. Several studies have independently confirmed the association of the *SIX1/SIX6* locus with both VCDR and POAG [7]–[9].

The human *SIX* gene family consists of six members (*SIX1*–*SIX6*), all of which contain two shared protein domains; a DNA binding homeobox domain and a SIX domain, which binds downstream effector molecules [10], [11]. Members of this conserved gene family were originally identified through homology to the *Drosophila melanogaster* (*Drosophila*) sine oculis (*so*) gene, which is required for proper eye development [10], [11] and are thought to function as transcription factors, regulating key developmental steps through a complex regulatory network. During embryonic development, *SIX1* is expressed broadly in multiple tissues, including the otic vesicle and the limb mesenchyme. However, expression of *SIX6* is restricted to regions of the retina and the pituitary [10], [12]. *Drosophila* with null *so* alleles have restricted retinal development, while morpholino knockdown of *six6b* in *Danio rerio* (zebrafish) embryos was recently shown to result in a small eye phenotype [10], [13]. In humans, a large deletion on chromosome 14q22.3-q23 that includes *SIX6* causes bilateral anophthalmia, the absence of both eyes, demonstrating the importance of *SIX* gene family members in ocular development and human disease [10], [11], [14]–[16].

In this study, we have extended the current understanding of the molecular contributions of *SIX6* to POAG risk. First, we identified potential POAG risk alleles by sequencing the *SIX6* gene in a case-control dataset; we found both common and rare coding changes within *SIX6* in POAG cases, as well as sequence variants in the *SIX6* enhancer. We then used the zebrafish system to demonstrate that these human coding variants have functional consequences in eye development. This analysis of 2–3 day old zebrafish embryos is not intended to fully recapitulate the glaucomatous phenotype; however, it provides *in vivo* data about the functional effects of human genetic variation on the human SIX6 protein in the context of eye development. We next used luciferase reporter assays, through which we show that a sequence variant found in the *SIX6* enhancer of POAG patients may increase *SIX6* expression. Finally, we demonstrate that POAG cases homozygous for the *SIX6* risk allele rs33912345 have a significantly thinner retinal nerve fiber layer, suggesting a glaucomatous pathogenic mechanism driven by SIX6 dysfunction.

## Results

### Identification of *SIX6* risk alleles in POAG cases

Sequencing of the *SIX1* and *SIX6* genes in Caucasian POAG cases and controls (262 cases, 256 controls) revealed 23 SNPs (Supplemental Table S1). Nine SNPs were identified in *SIX1*, but no nonsynonymous SNPs were present in the POAG cases. Sequencing of *SIX6* yielded 14 variants including five rare nonsynonymous SNPs in POAG cases and controls, one common nonsynonymous SNP located in the homeobox of *SIX6* (rs33912345, Asn141His), and five sequence variants within the *SIX6* enhancer. All of these variants are conserved evolutionarily as shown by their positive Genomic Evolution Rate Profiling (GERP) scores (Table 1) [17].

**Table 1:** *SIX6* variants identified by sequencing POAG cases and controls.

Gene	Coordinates	SNP ID	MAF POAG Cases	MAF POAG Controls	Base Change	AA Change	GERP
**Enhancer**	Chr14:60974363	----	0.002	0	G>C	----	3.77
**Enhancer**	Chr14:60974373	rs8004739	0.008	0	C>T	----	5.58
**Enhancer**	Chr14:60974378	----	0	0.002	C>T	----	2.77
**Enhancer**	Chr14:60974400	----	0.002	0.002	C>A	----	5.58
**Enhancer**	Chr14:60974449	----	0.002	0	A>G	----	3.48
***SIX6***	Chr14:60976393	rs78954112	0	0.002	G>C	Glu93Gln	5.52
***SIX6***	Chr14:60976501	rs146737847	0.008	0.002	G>A	Glu129Lys	5.38
***SIX6***	Chr14:60976537	rs33912345	0.47	0.37	A>C	Asn141His	5.38
***SIX6***	Chr14:60977843	rs45549246	0.002	0.002	T>G	Leu205Arg	4.14
***SIX6***	Chr14:60977864	rs202029915	0.002	0	C>T	Thr212Met	2.36
***SIX6***	Chr14:60977954	rs139302405	0.002	0	G>T	Ser242Ile	5.19

Coordinates are based on the Hg19 reference; MAF = minor allele frequency;

AA = amino acid; GERP = Genomic Evolutionary Rate Profiling.

Genotyping of rs33912345 in the Duke POAG case-control dataset (482 cases, 433 controls) resulted in a significant association (OR = 1.40, p = 0.0005, POAG case minor allele frequency (MAF) = 0.47, POAG control MAF = 0.38) with POAG. This SNP is in high linkage disequilibrium (r^2^ = 0.95) with the intergenic SNP identified originally in POAG and VCDR genome-wide association studies (rs10483727) [4]–[6], [13]. As expected, meta-analysis of the imputed genotype data from the NEIGHBOR and GLAUGEN studies confirmed a significant association between POAG status and rs33912345 (OR = 1.27, p = 4.2×10^−10^) and other linked SNPs in the region (Supplemental Table S2). Further examination of this locus showed that the association signal includes both upstream and downstream regions of the *SIX6* transcript, while remaining entirely downstream of *SIX1* (Supplemental 1).

We next performed optical coherence tomography (OCT) to study the retinal characteristics of POAG cases possessing the *SIX6* risk and non-risk variants (Table 2). OCT images were only available for POAG cases with the common SNP, rs33912345; no data were available for individuals with the rare *SIX6* variants. We assessed retinal nerve fiber layer (RNFL) thickness in thirty POAG cases homozygous for the rs33912345 risk allele (C) or the non-risk allele (A) first by comparing age at disease diagnosis and age at OCT across the two genotypes, because age is known to influence retinal thickness and is thus a potential confounder. We observed no significant difference in age (p = 0.11, p = 0.14, respectively). Next, RNFL thickness was evaluated. The overall thickness (global RNFL) was reduced significantly in cases homozygous for the risk allele compared to cases with the non-risk allele (p = 0.03; mean (SD): C = 58.3 (8.2) µm, A = 67.9 (12.4) µm; Table 2), consistent with the hypothesis that *SIX6* may increase POAG susceptibility via changes in the neural retina. To determine which quadrants might be driving this observation, we performed an exploratory, post-hoc comparison of RNFL thickness in the temporal, nasal, inferior, and superior regions. We found that RNFL thickness was reduced significantly in the inferior (p = 0.03) and superior (p = 0.04) quadrants, the two regions affecting directly VCDR measurements.

**Table 2:** OCT measurements from POAG cases homozygous for rs33912345.

Variable	Allele	N	Mean (SD)	p
Age at POAG diagnosis (years)	A	18	63.9 (9.1)	0.11
	C	12	69.7 (10.2)	
Age at OCT measurement (years)	A	18	71.2 (9.7)	0.13
	C	12	76.9 (10.3)	
Nerve fiber thickness (µm): Global	A	18	67.9 (12.4)	0.03
	C	12	58.3 (8.2)	
Temporal	A	18	57.2 (15.2)	0.85
	C	12	56.2 (12.8)	
Nasal	A	18	57.4 (13.4)	0.06
	C	12	47.9 (12.8)	
Inferior	A	18	79.6 (18.7)	0.03
	C	12	65.2 (12.6)	
Superior	A	18	77.0 (22.2)	0.04
	C	12	63.9 (10.8)	

SD = standard deviation; OCT = optical coherence tomography.

### *In vivo* functional interrogation of *SIX6* missense variants

Given 1) the observed association signal pattern; 2) the lack of coding changes identified in *SIX1*; 3) the presence of rare missense variants and a common, associated missense SNP in *SIX6*; 4) retinal nerve fiber layer thickness changes observed in POAG cases homozygous for the *SIX6* risk allele; and 5) the localized expression of *SIX6* in ocular tissues, we concluded *SIX6* is a likely candidate gene in this region. We therefore evaluated the functional relevance of *SIX6* and the potential burden of common and rare alleles in this locus in POAG using an *in vivo* zebrafish complementation assay.

First, we performed a reciprocal BLAST analysis; we identified two orthologs of SIX6 in the zebrafish genome, *Six6a* and *Six6b*, both with 91% homology at the protein level (Supplemental Figure S2). Previous overexpression and loss of function studies of *Six6* in *Mus musculus* (mouse) and *Xenopus laevis* (*Xenopus*) models reveal a role in regulating the proliferative state of retinal progenitor cells and the size of the eye [18], [19]; therefore, as a first test of whether the identified *SIX6* variants are pathogenic and potentially relevant to POAG, we asked whether 1) morpholino-induced suppression of *six6a* or *six6b* leads to a reduced eye size; 2) expression of the human *SIX6* non-risk allele rescues the morphant eye phenotype; and 3) expression of *SIX6* alleles containing POAG risk variants rescues the morphant eye phenotype.

**Figure 1:**
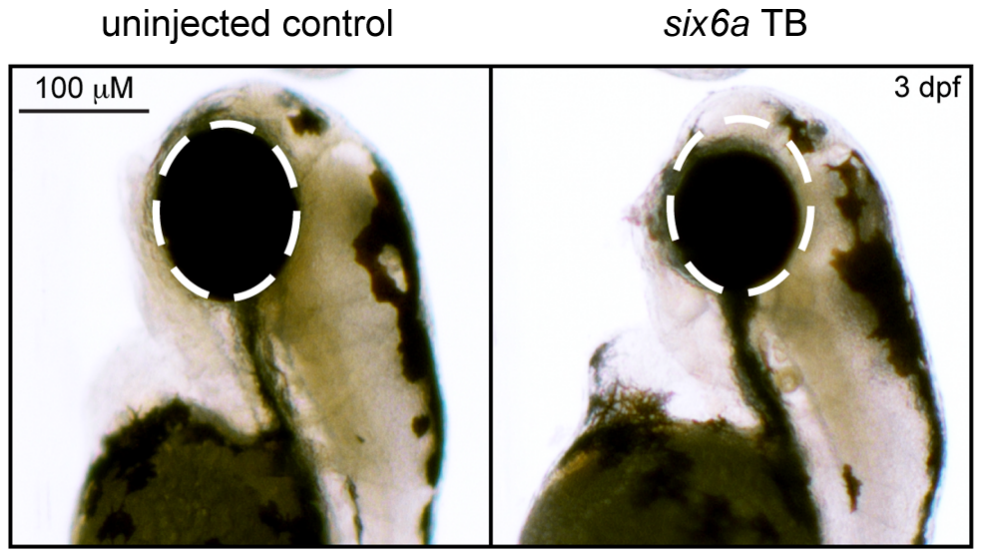
Morpholino knockdown of *six6a*. Zebrafish were microinjected with a *six6a* translation blocking morpholino. Lateral images, taken 3 days post fertilization (3 dpf), of a wild-type zebrafish (left) and a morpholino injected zebrafish (right) are shown, highlighting the small eye phenotype (dashed circle).

Using translation-blocking morpholinos (MOs) targeting zebrafish *six6a* and *six6b*, we injected 1–8 cell stage embryos (N = 50–150) and analyzed live embryos at 3 days post fertilization (dpf). We also tested a splice blocking MO. However, this induced non-specific toxicity, including the accumulation of pericardial fluid that could not be rescued with the human *SIX6* transcript. This is not unexpected, as *SIX6* is a two exon gene—splice-blocking MOs are generally not recommended for two exon genes; the targeted transcript will not be subject to nonsense mediated decay, possibly leading to the expression of a truncated protein and potential dominant-negative effects (described by the manufacturer: http://www.gene-tools.com/node/18). For these reasons, we used translation-blocking MOs for the remainder of our experiments. Masked scoring of both *six6a* and *six6b* morphants revealed ocular phenotypes consistent with loss of function, including a reduction in eye size in more than 80% of embryos (p<0.001, Figure 1). The specificity of the MO was tested by co-injection of 12.5 pg of the human *SIX6* non-risk allele mRNA; we observed significant (p<0.001) rescue in *six6a* but not *six6b* morphant embryos (90% vs. 10% of embryos, respectively). Together, these data indicate that Six6a is the functional ortholog of human SIX6 and prompted subsequent evaluation of *SIX6* variants using the *six6a* MO.

**Figure 2:**
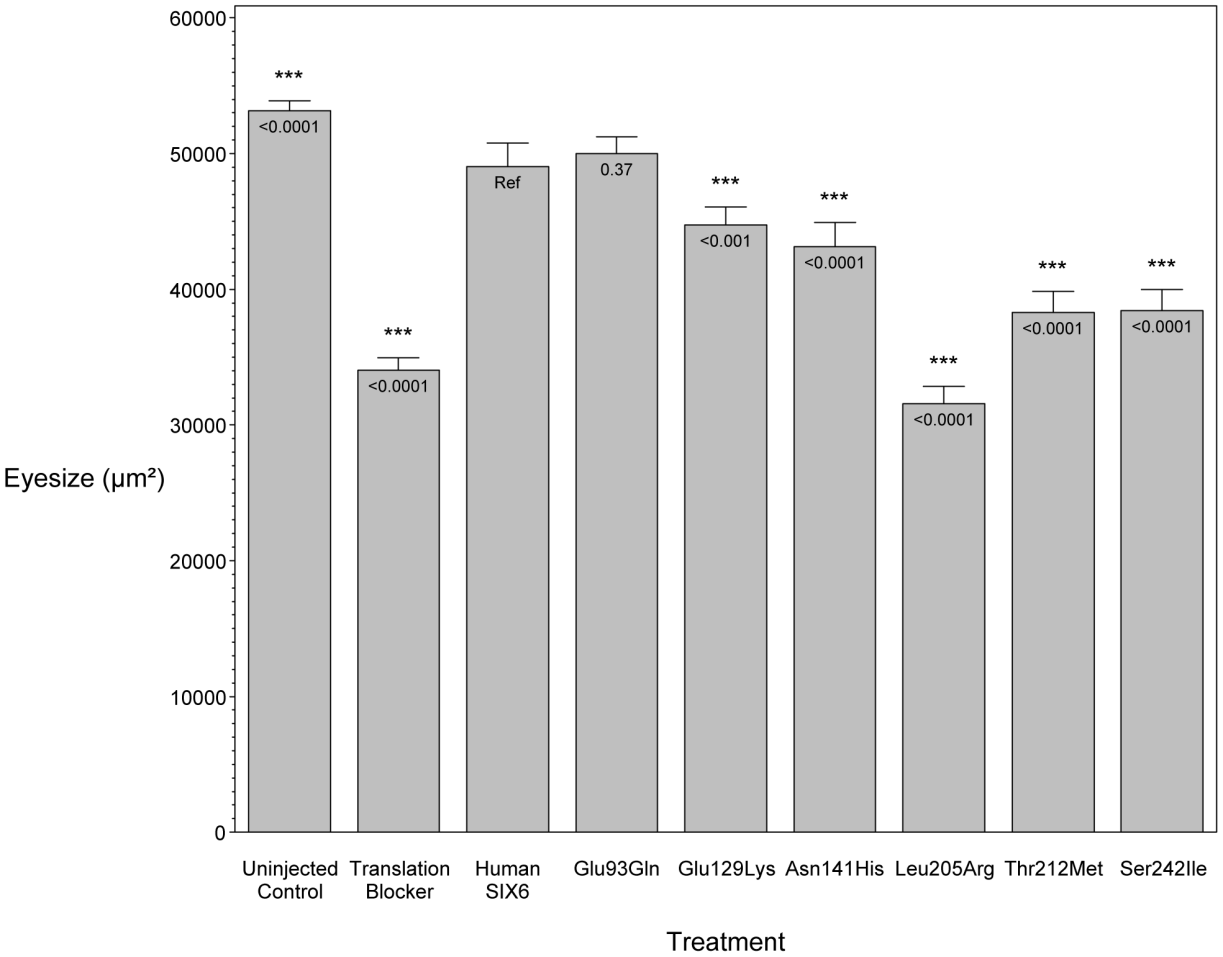
*In vivo* zebrafish morpholino complementation assay showing the effect of *SIX6* nonsynonymous variants. Zebrafish embryos were microinjected with a translation blocking morpholino designed to target *six6a*. Total eye size (µm^2^) was measured 3 days post fertilization. Compared to the uninjected controls, morphants showed a significant reduction in eye size. Zebrafish were co-injected with the morpholino and a human *SIX6* allele (Glu93Gln, Glu129Lys, Asn141His, Leu205Arg, Thr212Met, or Ser242IIe). Results of each allele were compared to the *SIX6* non-risk allele (Ref). P-values are provided below the mean of each treatment.

To investigate the pathogenic potential of all *SIX6* variants, we used total eye size and the rescue of the morphant phenotype as the assay's phenotypic readout. We injected a mixture containing *six6a* MO and each of the human *SIX6* alleles containing the coding variants identified via sequencing (Table 1). Subsequent to triplicate injections and masked scoring, these results were compared to the rescue condition of the human *SIX6* non-risk allele. We found that five of the six variants tested were unable to fully rescue the small eye phenotype. Four of these alleles (Glu129Lys, Asn141His, Thr212Met, and Ser242Ile) resulted in an average eye size larger than the morpholino alone (p<0.001), but smaller than the rescue with the non-risk allele (p<0.001), indicating that these alleles are hypomorphic (Figure 2, Table 3). We also observed one variant (Leu205Arg) with an average eye size smaller than the morpholino alone (p = 0.002; mean (SD): MO = 34,042 (5,763) µm^2^, Leu205Arg = 31,568 (6,485) µm^2^; Figure 2), suggesting that it is functionally null. Finally, one allele (Glu93Gln) resulted in an eye size similar to the rescue with the non-risk allele (p = 0.37) and was determined to be benign. The benign allele was identified in one control individual, while the remaining hypomorphic and null alleles were identified either exclusively or primarily in POAG cases (Table 1). Injection of 12.5 pg of the human *SIX6* risk mRNA into non-morphant zebrafish provided no evidence of a toxic gain of function compared to injection with the non-risk allele (data not shown).

**Table 3:** Results from the *in vivo* zebrafish *six6a* morpholino complementation assay.

Treatment		N	Mean (SD), µm^2^	p
Uninjected	Control	146	53,140 (4,536)	<0.0001
Translation Blocker	Morpholino	153	34,042 (5,763)	<0.0001
Human *SIX6*	Non-risk allele	177	49,040 (11,652)	Ref
Glu93Gln	Variant allele	92	50,010 (5,893)	0.37
Glu129Lys	Variant allele	100	44,719 (6,791)	<0.001
Asn141His	Variant allele	129	43,150 (10,660)	<0.0001
Leu205Arg	Variant allele	101	31,568 (6,485)	<0.0001
Thr212Met	Variant allele	84	38,278 (7,244)	<0.0001
Ser242IIe	Variant allele	88	38,420 (7,310)	<0.0001

N = sample size; SD = standard deviation.

**Figure 3:**
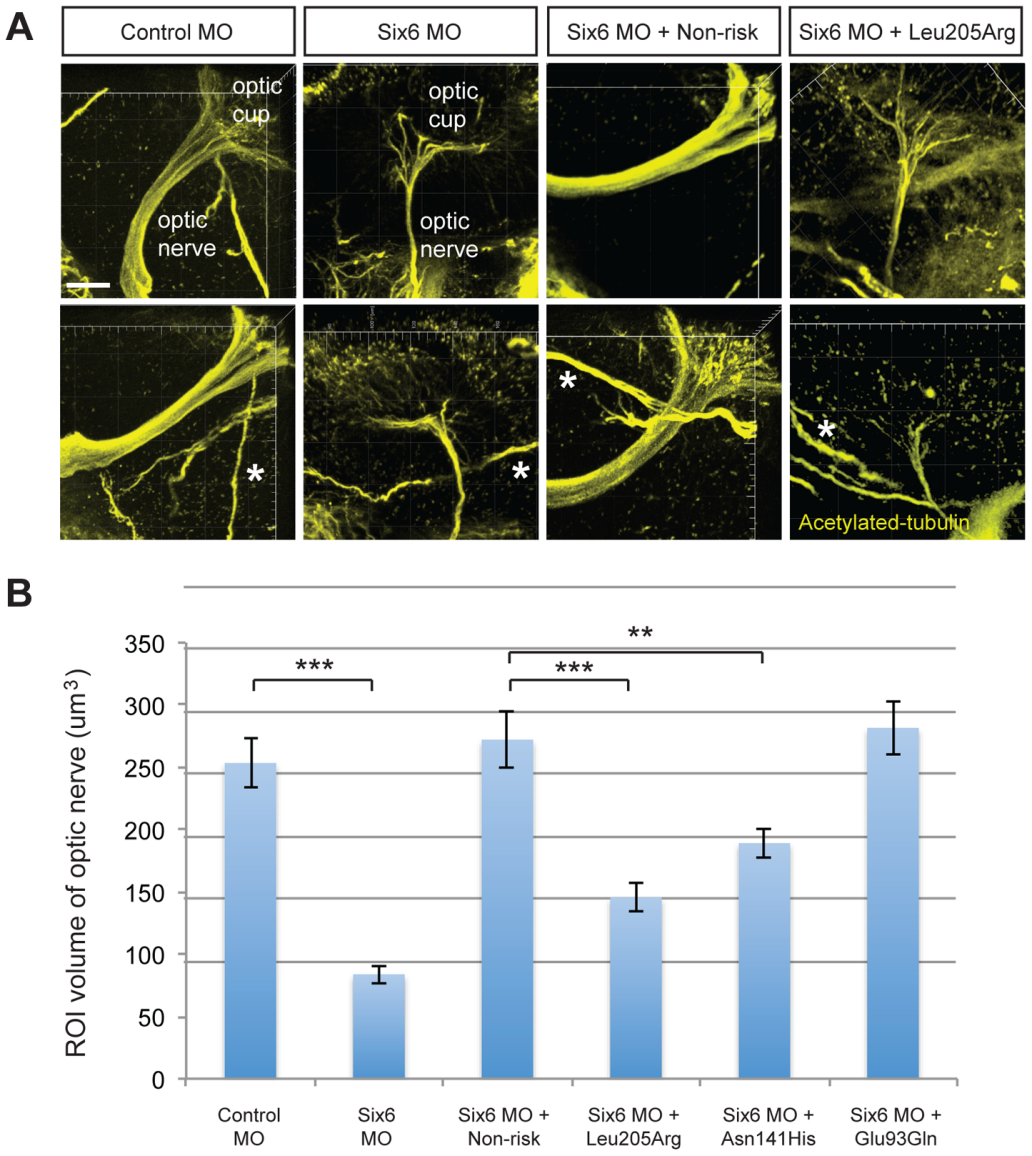
Functional evaluation of *SIX6* variants on the volume of the optic nerve. Representative whole mount images of acetylated-tubulin expression in the heads of zebrafish embryos injected with a control or *six6a* morpholino, rescued by co-injection with human non-risk SIX6 transcript or a transcript containing the Leu205Arg hypomorphic variant (A). Acetylated-tubulin staining is restricted primarily to axon tracts and can be used to visualize the optic nerve. Relative to the control morphants, volumetric regions of interest (ROI) along the optic nerve in *six6a* morphants were reduced significantly. Co-injection of human variants revealed a hypomorphic (Leu205Arg, Asn141His) or benign (Glu93Gln) role of the variants on the optic nerve (B). Sample size for all injection paradigms ranged from 7–9 and p-values are plotted for each comparison (*** p<0.001; ** p<0.01). No significant changes in the volume of other axonal tracts in the head (marked by an asterisk) were detected. Standard error of the mean is shown and white scale bars = 20 um.

Given the reduction of the RNFL in cases homozygous for the risk allele, we next asked whether *six6a* and the identified *SIX6* variants impacted the optic nerve, an anatomical site directly relevant to human POAG, in zebrafish. Using whole mount imaging of acetylated-tubulin expression in 2-dpf embryos injected with a control and *six6a* morpholino, we evaluated volumetric regions of interest (ROI) along the optic nerve (Supplemental Figure S3). Masked scoring of embryos revealed an approximately 3 fold reduction (p<0.001) in the volume of the optic nerve upon depletion of *six6a* (Figure 3 A–B). This was specific for the optic nerve as the volume of other axonal tracts in the brain were unaffected by *six6a* depletion (Figure 3A). Specificity of the volumetric measurements was demonstrated upon full rescue of the *six6a* morphant phenotype by co-injection of the non-risk allele (p<0.001) or a variant that scored as a benign allele in the eye size assay (Glu93Gln; p<0.001; Figure 3B). Both Leu205Arg and Asn141His variants performed as hypomorphic alleles (p<0.01), revealing concordance of our optic nerve assay with the OCT imaging findings in patients.

### *In vitro* functional interrogation of *SIX6* enhancer variants

**Figure 4:**
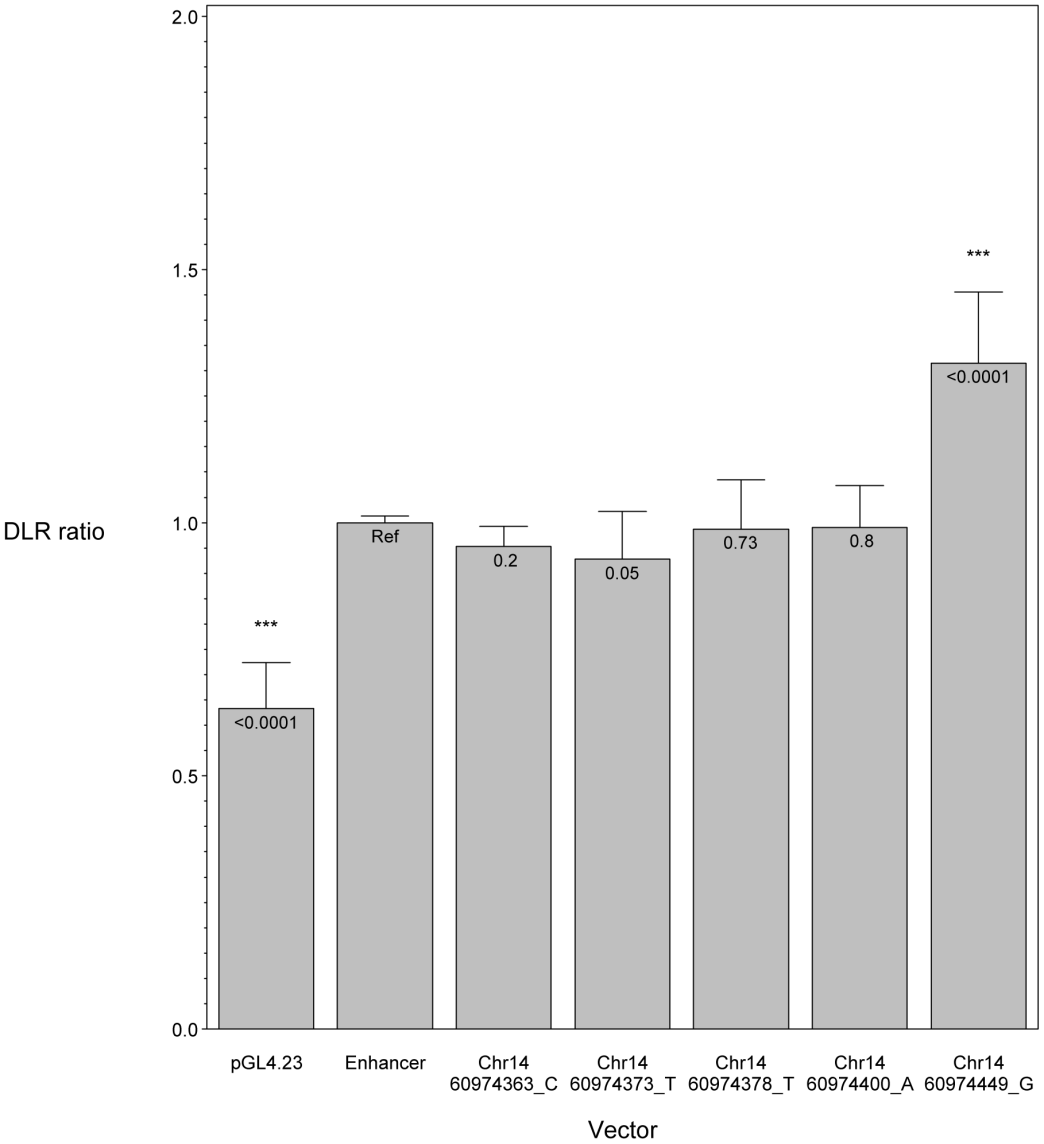
*In vitro* luciferase assay results showing the effect of *SIX6* enhancer variants. *SIX6* enhancer alleles were tested using a dual luciferase assay and the ratio of the experimental luciferase: control luciferase was calculated (DLR ratio). All vectors were co-transfected with NeuroD and E47. In this context, the *SIX6* enhancer is functioning to increase expression compared to the empty vector (pGL4.23), driven by a minimal promoter. Compared to the reference enhancer (Ref), one variant (Chr14:60974449_G) significantly increases the enhancer's activity.

We hypothesized that POAG risk may be mediated not only by deficits in SIX6 protein function, but also by the level of *SIX6* gene expression. To test this, we sequenced the *SIX6* retinal specific enhancer element in 262 POAG cases and 256 POAG controls; we identified five variants (Chr14:60974363_C, Chr14:60974373_T, Chr14:60974378_T, Chr14:60974400_A, Chr14:60974449_G) (Table 1), and tested their effect on expression using an *in vitro* luciferase assay. We found that one of these variants (Chr14:60974449_G) resulted in a significant increase in expression compared to the reference enhancer (Figure 4). Activation of the SIX6 enhancer requires two cofactors, NeuroD and E47 (Supplemental Figure S4) [12]. Overexpression was observed with the Chr14:60974449_G variant even in the absence of these cofactors (Supplemental Figure S5), suggesting variants within the enhancer region may result in dysregulated protein expression.

## Discussion

The *SIX1/SIX6* locus has been shown to be associated significantly with POAG in several independent studies; however, the causal variant(s) driving this association have remained unknown [4], [6]–[9], [13], as has the direction of effect of these common alleles on protein function. We have demonstrated through several lines of evidence—the tissue specificity of the *SIX* gene family described in the literature; the identification of *SIX6* missense variants in our POAG dataset; and the results of the *in vivo* and *in vitro* assays— that *SIX6* is the most likely POAG susceptibility gene in this region. We identified both common and rare coding variants that alter the function of the SIX6 protein. We also identified a novel variant within the *SIX6* enhancer that appears to disrupt the regulation of SIX6 expression, suggesting both regulatory and coding variants may influence POAG susceptibility at this locus. Finally, we identified hitherto unknown clinical features in POAG patients that may be dependent upon *SIX6* genotype: patients homozygous for the *SIX6* risk allele have a significantly thinner retinal nerve fiber layer than patients homozygous for the *SIX6* non-risk allele.

The common variant, rs33912345 (Asn141His), which we showed has significantly reduced function in an *in vivo* model, was associated with POAG in our dataset and is in strong linkage disequilibrium with the originally identified GWAS SNP, rs10483727 [4]. This conserved amino acid is located within the alpha helix structure of the DNA homeobox domain of *SIX6*. Interestingly, the ancestral allele (C, His) is associated with POAG risk. The allele frequency of this variant differs markedly among populations (C allele frequency: YRI (0.99), ARF (0.96), ASN (0.76), and CEU (0.42); from 1000 Genomes release 14) [20]. This locus is associated significantly with an increase in vertical cup-disc ratio (VCDR) in population controls, indicating that it may be involved in the development of the optic nerve. VCDR is also a clinical measure used to track disease progression in POAG patients [5], [6], [9], [13], [21]. We note that African populations have larger VCDR and an increase in overall POAG prevalence [22]–[24] compared to CEU populations; in a recent study, the prevalence of POAG in Ghana exceeded 17%, the highest rate observed anywhere in the world [25]. While rs33912345 is not associated with POAG risk in a West African POAG case/control dataset from Ghana, the frequency of the ancestral (risk) allele is 99% in both cases and controls [26]. We hypothesize that differences in the structure of the optic nerve and the higher risk of POAG in individuals with African ancestry may, in part, arise from the fact that essentially all individuals in this population are homozygous for the rs33912345 ancestral risk allele.

Given the association of the *SIX6* locus with neural retinal measurements such as VCDR, it is reasonable to hypothesize causal variants may function by inducing quantitative changes in cell populations in the retina. There is extensive evidence that SIX6 regulates early retinal progenitor cell proliferation during eye development [18], [19], [27]. Li *et al.* showed that *Six6* ^−/−^ mice display varying degrees of retinal hypoplasia that is due to a decrease in retinal ganglion cell proliferation arising from an early exit from the cell cycle during development, and results in a 20% decrease in the number of retinal ganglion cells by P35 [18]. This is consistent with functional studies of *XOptx2*, the *Xenopus* ortholog of *SIX6* [19]. We have shown a reduction in eye size and in the volume of the optic nerve upon MO knockdown of zebrafish *six6a*, and we were able to rescue these phenotypes with co-injection of the human *SIX6* non-risk allele, demonstrating that the zebrafish *six6a* gene is the likely functional ortholog to human *SIX6*. We identified five alleles that could not rescue the small eye and optic nerve phenotypes, and we observed a reduction in retinal nerve fiber layer thickness in POAG patients homozygous for the His141 SIX6 risk allele. Taken together with previously published findings, our results suggest that risk variants in human *SIX6* increase POAG susceptibility by negatively affecting retinal ganglion cell development, likely leading to a reduction in the number of retinal ganglion cells in adulthood. Given that retinal ganglion cells are lost during the normal aging process, we speculate that this rate of loss could be increased by the presence of additional POAG risk alleles or other risk factors such as increased intraocular pressure [28]. The development of glaucomatous optic neuropathy and associated visual field loss would thus be hastened by a reduction in the initial number of retinal ganglion cells that an individual possesses. Future work will test the possibility that SIX6 variants also alter the rate of RGC death in the adult.

In summary, we have identified multiple common and rare *SIX6* sequence variants in POAG cases, and used *in vivo* and *in vitro* assays to demonstrate that these variants have functional consequences on *SIX6* expression and protein function. While other risk factors may be required for the onset of POAG, our data suggest that attenuation of SIX6 protein function increases an individual's susceptibility to developing the disease via changes to retinal development. Additional work is needed, possibly through the use of transgenic animal model studies, to fully understand the role of SIX6 in POAG.

## Materials and Methods

### Ethics statement

This research was approved by the Institutional Review Board of Duke University Medical Center and adheres to the tenets of the Declaration of Helsinki.

### Subjects

Study subjects were unrelated patients from the Duke Eye Center and, after a comprehensive eye examine, were classified as either POAG cases or controls. POAG cases presented with glaucomatous optic neuropathy, defined as a cup-to-disc ratio greater than 0.7 and visual field loss in at least one eye. Patients with secondary forms of glaucoma or a history of ocular trauma were excluded from the study. POAG controls had no evidence of optic neuropathy, normal intraocular pressure (less than 22 mmHg in both eyes), and normal visual fields, assessed using standard automated perimetry.

### DNA sequencing

Genomic DNA was extracted from patient blood samples using the PureGene chemistry following the manufacturer's standard protocol (Gentra, Minneapolis, MN). The coding portions (2 exons) of the *SIX1* and *SIX6* genes were sequenced in 518 Caucasian POAG cases and controls (262 cases, 256 controls) using a polymerase chain-reaction (PCR) containing 1× Qiagen PCR buffer (Tris·Cl, KCl, (NH4)2SO4, 15 mM MgCl2; pH 8.7); 200 µM each of dATP, dCTP, dGTP, and dTTP; 0.4 µM forward PCR primer; 0.4 µM reverse PCR primer; 3 µL of betaine, 10 ng genomic DNA; and 0.5 U HotStarTaq DNA polymerase (Qiagen, Venlo, Limburg) to a final volume of 25 µL. Primer sequences are available in Supplemental Table S3. The PCR was performed using a touchdown protocol (incremental lowering of annealing temperature) using the following thermocycler conditions: 94°C for 30 s, 65°C for 30 s, 72°C for 30 s with a 2°C decrease in the annealing temperature every two cycles until a final annealing temperature of 55°C was reached. The retinal specific *SIX6* enhancer, previously described [12], was amplified a using a touchdown protocol with a final annealing temperature of 57°C. PCR products were sequencing using the BigDye chemistry on a 3730 DNA Analyzer (Applied Biosystems, Grand Island, NY).

### Genotyping and imputation

The common missense single nucleotide polymorphism (SNP), rs33912345, was genotyped in the Duke POAG case-control dataset consisting of 482 POAG cases and 433 POAG controls using a TaqMan allelic discrimination assay according to the standard protocols from the manufacturer (Life Technologies, Grand Island, NY). For quality control purposes the following criteria were met: >95% genotyping efficiency, matching sample duplicates (two Centre d'Etude du Polymorphisme Humain samples per 96-well plate whose genotype data matched across all plates), and Hardy-Weinberg equilibrium assumptions. We tested for association of rs33912345 with POAG using an additive logistic regression model adjusted for age and sex using SAS [29].

Genome-wide genotype data were available from the NEI Glaucoma Human Genetics Collaboration (NEIGHBOR) and the Glaucoma Genes and Environment (GLAUGEN) consortia [4]. Chromosome 14 was imputed using IMPUTE2 (http://mathgen.stats.ox.ac.uk/impute/impute_v2.html) with a global 1000 Genomes reference panel. We tested SNPs at the *SIX1/SIX6* locus for association with POAG using an additive logistic regression model adjusted for age, sex, and four principal components (NEIGHBOR) or age, gender, study site, DNA extraction method, DNA specimen type and principal components 1–6 (GLAUGEN) implemented in PLINK and visualized using LocusZoom (http://csg.sph.umich.edu/locuszoom/) [30], [31]. A meta-analysis was performed in Plink using a random effects model. Linkage disequilibrium was calculated and visualized using Haploview (http://www.broadinstitute.org/scientific-community/science/programs/medical-and-population-genetics/haploview/haploview) [32].

### Retinal nerve fiber layer thickness analysis

Optical coherence tomography (OCT) measurements of retinal nerve fiber layer (RNFL) by Spectralis (Heidelberg Engineering, Carlsbad, CA) spectral domain) and fundus photography were available from the Duke Eye Center. OCT images are not routinely performed in patients without ocular disease, so there was limited data available for controls. Therefore, the analysis was restricted to POAG cases homozygous for rs33912345. Thirty patients had both OCT measurements and *SIX6* genotype data. RNFL thickness, age at POAG diagnosis, and the age at OCT measurement were compared between individuals homozygous for the risk or non-risk allele using a Student's t-test. Analyses were performed in SAS [29].

### Microinjection of morpholino and mRNA

A vector containing human *SIX6* was purchased from the CCSB Human ORFeome Collection that uses the Gateway technology system (Open Biosystems and Life Technologies, Grand Island, NY). *SIX6* alleles identified by sequencing (Glu93Gln, Glu129Lys, Asn141His, Leu205Arg, Thr212Met, Ser242IIe), were created using the QuikChange II site-directed mutagenesis kit and protocols provided by the manufacturer (Agilent Technologies). *SIX6* mRNA was *in vitro* transcribed using mMESSAGE mMACHINE SP6 Kit (Ambion, Life Technologies, Grand Island, NY). Translation blocker (TB) morpholinos against *six6a* (5′- CTGGAACATGGAGACTGTAATGTCT -3′) and *six6b* (5′ AATTGGCAACTGAAACATGAAGGCT 3′) were purchased from Gene Tools, LLC. Morpholino (2 ng) and mRNA (12.5 pg) were mixed and a volume of 0.5 nL was microinjected into each wild-type zebrafish embryo at one- to eight-cell stage as described previously (Stuart, McMurray et al. 1988).

### Morphometric analyses of zebrafish

Morphometric analyses of eye size were conducted on zebrafish embryos at 3 days post fertilization, using a Nikon SMZ 745T microscope. Zebrafish were anesthetized in embryo medium containing 0.2 mg/ml tricaine (Ethyl 3-aminobenzoate methanesulfonate, Sigma, E10521). Lateral view images were captured with Nikon DS-Fi1 camera, and the size of eye was measured with Nikon NIS-Elements AR software. Analysis of the optic nerve was performed on 2 dpf embryos fixed in Dent's fixative (80% Methanol and 20% DMSO) overnight and stained with acetylated-tubulin (Sigma; T7451). Heads were isolated from stained embryos and oriented with the ventral aspect facing a coverslip on microscope cover glass. Image acquisition was performed on a Zeiss 710 inverted confocal microscope and ∼100 um optical sections were obtained and reconstructed. Volumetric measurements were calculated using Imaris software and 7.5 um×7.5 um×15 um ROIs along the optic nerve were analyzed between each condition. ROIs were restricted to portions of the optic nerve wherein all neurite processes coalesced to form the major aspect of the nerve.

### *In vitro* luciferase assay

*SIX6* enhancer alleles were tested using the dual-luciferase reporter assay system (Promega, Madison, WI). An experimental construct containing a minimal promoter (pGL4.23, firefly luciferase, Promega) was used to test the functional effect of the enhancer alleles identified by sequencing (Chr14:60974363_C, Chr14:60974373_T, Chr14:60974378_T, Chr14:60974400_A, Chr14:60974449_G) in the POAG case/control dataset. The experimental constructs (pGL4.23+Enhancer) were generated using a nested PCR protocol; the XhoI and HindII enzymes; the Quick Ligation kit (New England BioLabs, Ipswich, MA); and the QuikChange II site-directed mutagenesis kit (Agilent Technologies), following protocols provided by the manufacturers. Constructs were confirmed to be correct by sequencing. Hek293 cells were cultured according to the supplier's suggestions (ATCC, Manassas, VA). As described by Conte et al., co-transfection with *NeuroD* and *E47* is required for *SIX6* enhancer activation [12]. Therefore, cells were co-transfected with an experimental vector (pGL4.23+Enhancer), a control vector (pGL4.74, renilla luciferase, Promega), and vectors containing *NeuroD* and *E47* (provided by the Center for Human Disease Modeling, Duke University) using a standard calcium phosphate transfection protocol. The experiment was performed three times in triplicate and the results were analyzed using the dual luciferase reporter (DLR) ratio (firefly luciferase sum: renilla luciferase sum) normalized by the reference *SIX6* enhancer included on every plate. The data were analyzed using an ANOVA, adjusted for batch, and linear contrasts were used to determine the effect of each vector. Statistical analyses were performed in SAS [29].

## Supporting Information

Figure S1 Plot of the association signal from the NEIGHBOR and GLAUGEN POAG meta-analysis results observed at the SIX1/SIX6 locus calculated using chromosome 14 imputed genotype data. Generated using LocusZoom (http://genome.sph.umich.edu/wiki/LocusZoom). The color indicates the r^2^ value with the index SNP, rs10483727.
(TIF)

Figure S2 Results from BLAST search showing homology between human SIX6 and zebrafish Six6a and Six6b. The human SIX6 protein shares 91% identity with both of the zebrafish orthologs, Six6a and Six6b. Red: conserved across all proteins; Blue: conserved across 2 proteins; Grey: not conserved.
(TIF)

Figure S3 Volumetric analysis of the optic nerve. Confocal image of a 2 dpf zebrafish head stained with antibody to acetylated tubulin to visualize axons. (A) Regions of Interest (ROIs) 7.5 um×7.5 um×15 um in size along the optic nerve were selected from reconstructed 3-dimensional images using Imaris software [X–Y plane (B–C) and X–Z plane (D–E)]. Panels C and E show the reconstructed ROI from which volumetric measurements are calculated.
(TIF)

Figure S4 *In vitro* luciferase assay results from the *SIX6* enhancer and co-transfection with NeuroD and E47. The *SIX6* enhancer alone was inactive compared to the empty vector (pGL4.23). Co-transfection of both NeuroD and E47 increased the enhancer's activity. The highest activity level was reach with co-transfection of an equal amount of both, as previously described [12].
(PDF)

Figure S5 *In vitro* luciferase assay results showing the effect of the *SIX6* enhancer without co-transfection of NeuroD and E47. *SIX6* enhancer alleles were tested using a dual luciferase assay and the ratio of the experimental luciferase: control luciferase was calculated (DLR ratio). In the absence of NeuroD and E47, the *SIX6* enhancer is not active. However, the Chr14:60974449_G variant still shows increased enhancer activity. Coordinates are based on the Hg19 reference. P-values are provided below the mean of each vector.
(PDF)

Table S1 Coding variants identified by sequencing *SIX1* and *SIX6* in 518 POAG cases and controls. Coordinates are based on the Hg19 reference.
(DOCX)

Table S2 Top SNPs from the imputed Chromosome 14 POAG association analysis. Plink output from meta-analysis of the NEIGHBOR and GLAUGEN logistic regression results. OR = odds ratio.
(DOCX)

Table S3 Primer sequences used in this study.
(DOCX)
