# Supplementary figures and images for: Discovery and Functional Annotation of SIX6 Variants in Primary Open-Angle Glaucoma
Source: PLoS Genet. 2014 May 29;10(5):e1004372. doi: 10.1371/journal.pgen.1004372 (PMC4038608; doi:10.1371/journal.pgen.1004372)

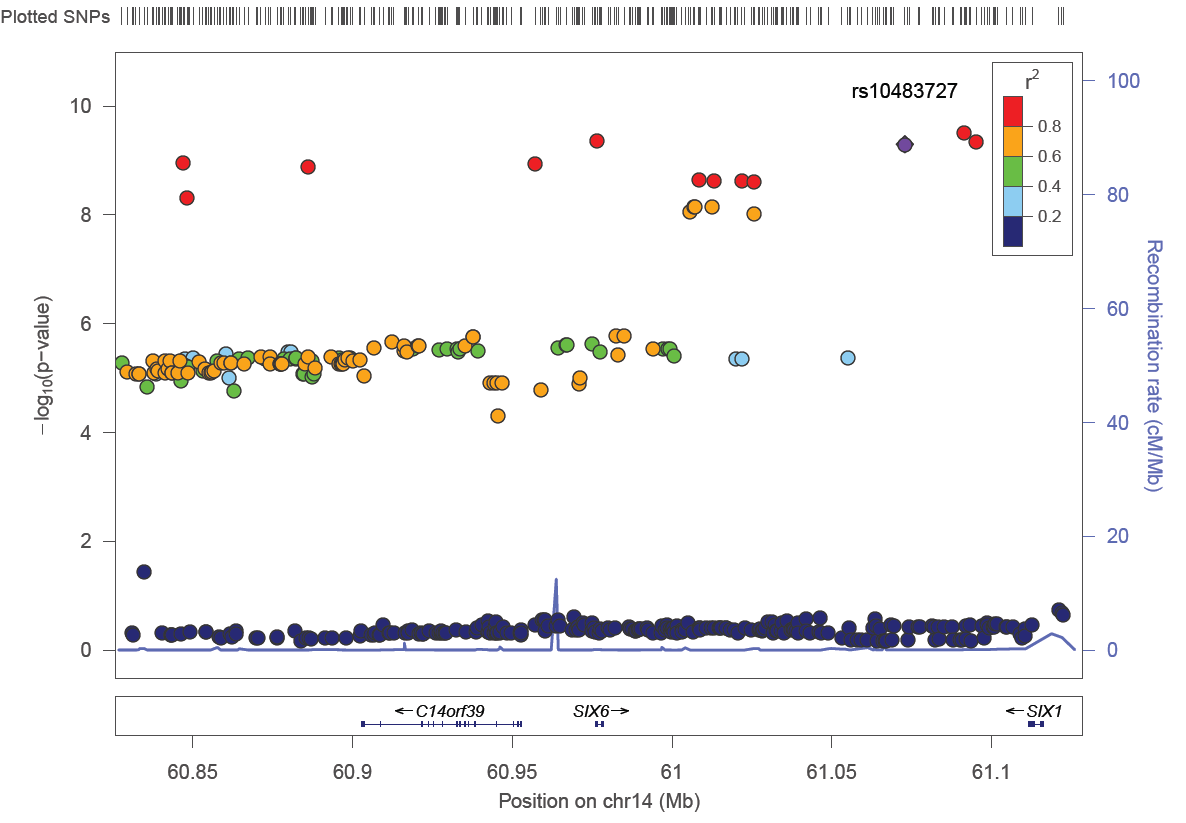

Supplement: Figure S1 — Plot of the association signal from the NEIGHBOR and GLAUGEN POAG meta-analysis results observed at the SIX1/SIX6 locus calculated using chromosome 14 imputed genotype data. Generated using LocusZoom (http://genome.sph.umich.edu/wiki/LocusZoom). The color indicates the r2 value with the index SNP, rs10483727. (TIF) [file pgen.1004372.s001.tif]

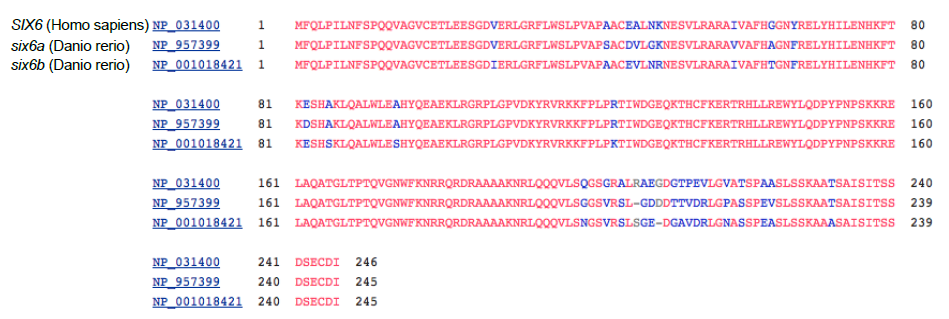

Supplement: Figure S2 — Results from BLAST search showing homology between human SIX6 and zebrafish Six6a and Six6b. The human SIX6 protein shares 91% identity with both of the zebrafish orthologs, Six6a and Six6b. Red: conserved across all proteins; Blue: conserved across 2 proteins; Grey: not conserved. (TIF) [file pgen.1004372.s002.tif]

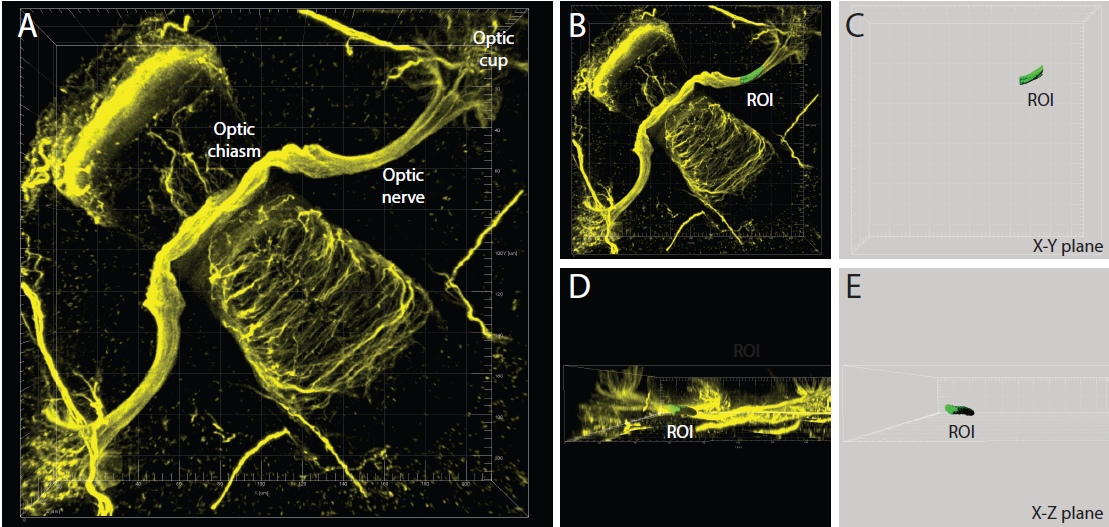

Supplement: Figure S3 — Volumetric analysis of the optic nerve. Confocal image of a 2 dpf zebrafish head stained with antibody to acetylated tubulin to visualize axons. (A) Regions of Interest (ROIs) 7.5 um×7.5 um×15 um in size along the optic nerve were selected from reconstructed 3-dimensional images using Imaris software [X–Y plane (B–C) and X–Z plane (D–E)]. Panels C and E show the reconstructed ROI from which volumetric measurements are calculated. (TIF) [file pgen.1004372.s003.tif]

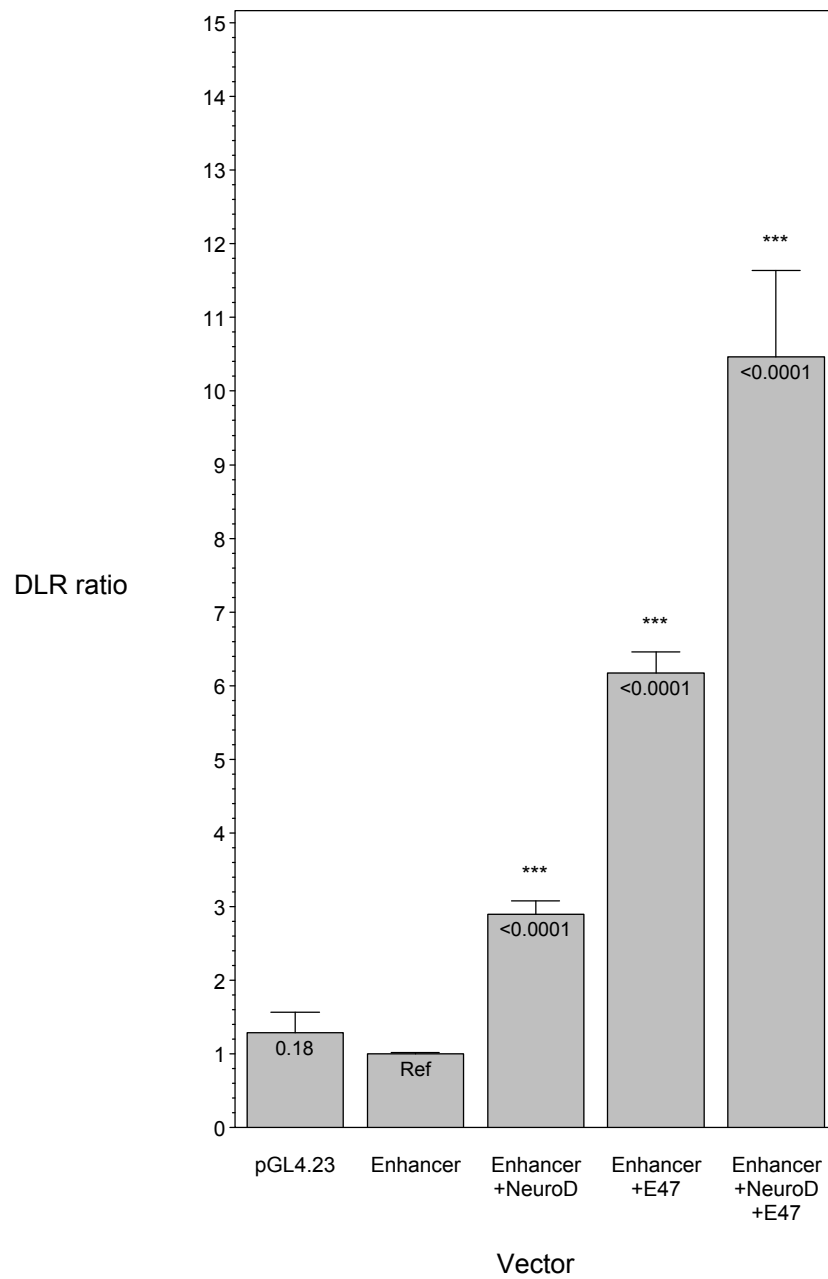

Supplement: Figure S4 — In vitro luciferase assay results from the SIX6 enhancer and co-transfection with NeuroD and E47. The SIX6 enhancer alone was inactive compared to the empty vector (pGL4.23). Co-transfection of both NeuroD and E47 increased the enhancer's activity. The highest activity level was reach with co-transfection of an equal amount of both, as previously described [12]. (PDF) [file pgen.1004372.s004.pdf]

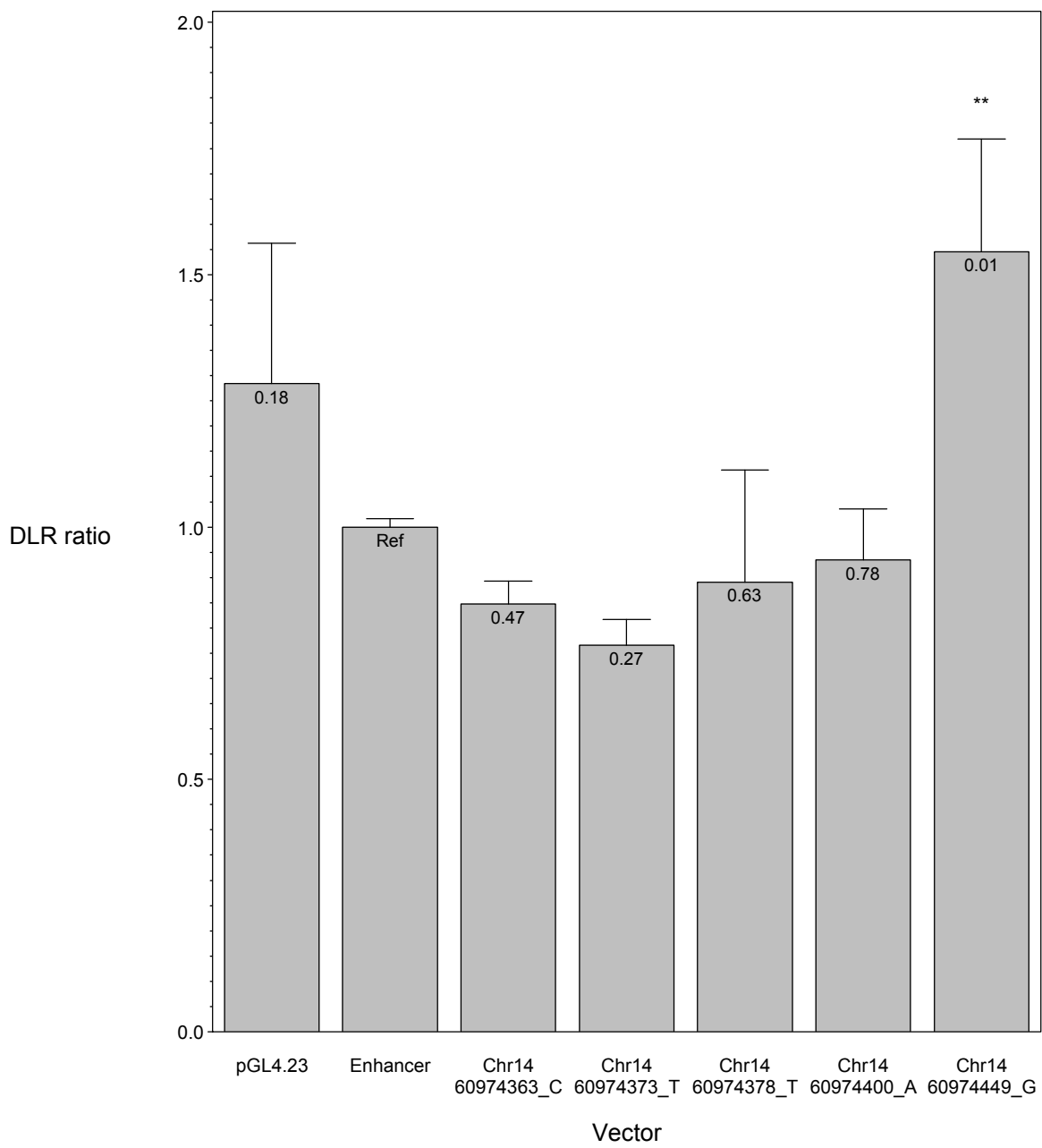

Supplement: Figure S5 — In vitro luciferase assay results showing the effect of the SIX6 enhancer without co-transfection of NeuroD and E47. SIX6 enhancer alleles were tested using a dual luciferase assay and the ratio of the experimental luciferase: control luciferase was calculated (DLR ratio). In the absence of NeuroD and E47, the SIX6 enhancer is not active. However, the Chr14:60974449_G variant still shows increased enhancer activity. Coordinates are based on the Hg19 reference. P-values are provided below the mean of each vector. (PDF) [file pgen.1004372.s005.pdf]
