# Supplementary material for: Discovery and Functional Annotation of SIX6 Variants in Primary Open-Angle Glaucoma
Source: PLoS Genet. 2014 May 29;10(5):e1004372. doi: 10.1371/journal.pgen.1004372 (PMC4038608; doi:10.1371/journal.pgen.1004372)
Supplement: Table S1 — Coding variants identified by sequencing SIX1 and SIX6 in 518 POAG cases and controls. Coordinates are based on the Hg19 reference. (DOCX) [file pgen.1004372.s006.docx]

| Gene | Coordinates | SNP ID | Function | Base Change | AA Change | Sample  Phenotype |
| --- | --- | --- | --- | --- | --- | --- |
| *SIX1* | Chr14:61115933 | ͞ | 5’ UTR | G>T | ͞ | Control |
| *SIX1* | Chr14:61115932 | ͞ | 5’ UTR | C>T | ͞ | Control |
| *SIX1* | Chr14:61115506 | rs151189392 | Synonymous | C>T | Lys134Lys | POAG |
| *SIX1* | Chr14:61115323 | rs200511291 | Intronic | C>G | ͞ | POAG |
| *SIX1* | Chr14:61115322 | rs374507275 | Intronic | G>C | ͞ | POAG |
| *SIX1* | Chr14:61113320 | ͞ | Intronic | delT | ͞ | POAG |
| *SIX1* | Chr14:61113316 | rs183396626 | Intronic | G>A | ͞ | Control |
| *SIX1* | Chr14:61113278 | rs142301715 | Missense | A>T | Asn193Ile | Control |
| *SIX1* | Chr14:61113110 | rs368974927 | Missense | G>T | Pro249Leu | Control |
| *SIX6* | Chr14:60976053 | rs148591528 | 5' UTR | C>T | ͞ | Control |
| *SIX6* | Chr14:60976137 | rs61746410 | Synonymous | G>A | Leu7Leu | Both |
| *SIX6* | Chr14:60976290 | rs45549246 | Synonymous | C>T | Ala58Ala | Control |
| *SIX6* | Chr14:60976393 | rs78954112 | Missense | G>C | Glu93Gln | Control |
| *SIX6* | Chr14:60976501 | rs146737847 | Missense | G>A | Glu129Lys | Both |
| *SIX6* | Chr14:60976537 | rs33912345 | Missense | A>C | Asn141His | Both |
| *SIX6* | Chr14:60977618 | rs56098605 | Intronic | G>A | ͞ | Both |
| *SIX6* | Chr14:60977756 | ͞ | Intronic | C>A | ͞ | POAG |
| *SIX6* | Chr14:60977774 | ͞ | Intronic | T>C | ͞ | POAG |
| *SIX6* | Chr14:60977843 | rs45549246 | Missense | T>G | Leu205Arg | Both |
| *SIX6* | Chr14:60977864 | rs202029915 | Missense | C>T | Thr212Met | POAG |
| *SIX6* | Chr14:60977954 | rs139302405 | Missense | G>T | Ser242Ile | POAG |
| *SIX6* | Chr14:60977955 | rs143366401 | Synonymous | C>T | Ser242Ser | POAG |
| *SIX6* | Chr14:60978071 | rs1061108 | 3’ UTR | G>C | ͞ | Both |
