# Supplementary material for: Discovery and Functional Annotation of SIX6 Variants in Primary Open-Angle Glaucoma
Source: PLoS Genet. 2014 May 29;10(5):e1004372. doi: 10.1371/journal.pgen.1004372 (PMC4038608; doi:10.1371/journal.pgen.1004372)
Supplement: Table S2 — Top SNPs from the imputed Chromosome 14 POAG association analysis. Plink output from meta-analysis of the NEIGHBOR and GLAUGEN logistic regression results. OR = odds ratio. (DOCX) [file pgen.1004372.s007.docx]

| BP | SNP | P | OR |
| --- | --- | --- | --- |
| Chr14:61091401 | rs34935520 | 3.07E-10 | 1.27 |
| Chr14:60976537 | rs33912345 | 4.20E-10 | 1.27 |
| Chr14:61095174 | rs35155027 | 4.39E-10 | 1.27 |
| Chr14:61072875 | rs10483727 | 5.02E-10 | 1.26 |
| Chr14:60811999 | rs8015152 | 8.61E-10 | 1.27 |
| Chr14:60847001 | rs1254276 | 1.10E-09 | 1.26 |
| Chr14:60957279 | rs2093210 | 1.12E-09 | 1.26 |
| Chr14:60813416 | rs10151339 | 1.25E-09 | 1.27 |
| Chr14:60886150 | rs1272131 | 1.29E-09 | 1.26 |
| Chr14:61008596 | rs7159392 | 2.22E-09 | 1.25 |
| Chr14:61013237 | rs2351174 | 2.36E-09 | 1.25 |
| Chr14:61021891 | rs1955695 | 2.36E-09 | 1.25 |
| Chr14:61025617 | rs4442732 | 2.44E-09 | 1.25 |
| Chr14:60848224 | rs1313237 | 4.78E-09 | 1.25 |
| Chr14:61006889 | rs12883754 | 7.05E-09 | 1.26 |
| Chr14:61007104 | rs10146342 | 7.05E-09 | 1.26 |
| Chr14:61012559 | rs12589689 | 7.05E-09 | 1.26 |
| Chr14:61005625 | rs1010053 | 8.66E-09 | 1.25 |
| Chr14:60789176 | rs4901977 | 9.27E-09 | 1.25 |
| Chr14:61025791 | rs1555211 | 9.34E-09 | 1.25 |
