# Supplementary material for: Discovery and Functional Annotation of SIX6 Variants in Primary Open-Angle Glaucoma
Source: PLoS Genet. 2014 May 29;10(5):e1004372. doi: 10.1371/journal.pgen.1004372 (PMC4038608; doi:10.1371/journal.pgen.1004372)
Supplement: Table S3 — Primer sequences used in this study. (DOCX) [file pgen.1004372.s008.docx]

| Amplicon | Forward Primer | Reverse Primer |
| --- | --- | --- |
| *SIX1* exon1 | 5’ TTGCAAAGCCTAAGGAGGAG 3’ | 5’ AGGACTTGGTGGCTGGTG 3’ |
| *SIX1* exon 2 | 5’ TTTGGGTTGGTGACAGATTG 3’ | 5’ GTCCACCATTCCTTTATGCG 3’ |
| *SIX6* exon 1 | 5’ ATAGTCCTGGCGTGCTGATT 3’ | 5’ CAGAACGCAGGGCTCTTAAC 3’ |
| *SIX6* exon 2 | 5’ TCCCAAAAGTGCACAACAAA 3’ | 5’ TTCCGAAGGAGACTTTGCAG 3’ |
| *SIX6* enhancer 1 | 5’ CGAGTGAACTGTGAAGATCTGTG 3’ | 5’ AGGTGAGAACGTTCACAGCCGA 3’ |
| *SIX6* enhancer 2 | 5’ GGGGCTCGAGCGAGTGAACTGTGAAG 3’ | 5’ GGGGAAGCTTAGGTGAGAACGTTCAC 3’ |
